# Safe and Stable Germline Transmission of MSTN Mutations in Cattle

**DOI:** 10.1002/age.70192

**Published:** 2026-08-30

**Authors:** Gyeong‐Min Gim, Bae Young Choi, Jeongbin Yi, Kyeong‐Hyeon Eom, Soo‐Young Yum, Dae‐Jin Jung, Do‐Yoon Kim, Bokyoung Yang, Jong‐Seo Kim, Donghwan Shim, Goo Jang

**Affiliations:** ^1^ LARTBio Inc. Seoul Republic of Korea; ^2^ School of Liberal Arts and Sciences Korea National University of Transportation Chungju Republic of Korea; ^3^ Center for RNA Research Institute for Basic Science Seoul Korea; ^4^ School of Biological Sciences Seoul National University Seoul Korea; ^5^ Gyeongsangbukdo Livestock Research Institute Yeongju Republic of Korea; ^6^ Theragen Bio Co. Ltd. Seongnam‐si Gyeonggi‐do Republic of Korea; ^7^ Department of Biological Sciences Chungnam National University Daejeon Republic of Korea; ^8^ Center for Genome Engineering Institute for Basic Science Daejeon Republic of Korea; ^9^ Laboratory of Theriogenology and Biotechnology, Department of Veterinary Clinical Science College of Veterinary Medicine and the Research Institute of Veterinary Science, Seoul National University Seoul Republic of Korea

**Keywords:** food security, genome stability, germline transmission, *MSTN* mutated cattle

## Abstract

With the global population expected to reach 10 billion by 2050, sustainable livestock production is critical. Gene editing of the myostatin (MSTN) gene represents a promising strategy to enhance muscle growth in cattle. In this study, MSTN‐mutated founder (F0) cows were used to generate F1 offspring via ovum pick‐up, in vitro fertilization, and embryo transfer. Four F1 calves were born, all confirmed to be heterozygous for the MSTN mutation. Long‐term monitoring showed normal growth and no visible health abnormalities. Whole‐genome sequencing identified SNPs, INDELs, and structural variants, most with minimal predicted functional effects. Proteomic profiling of Longissimus dorsi muscle quantified 2947 proteins, revealing only subtle expression differences between MSTN‐mutated and wild‐type cattle. These results demonstrate stable inheritance and confirm that MSTN editing does not disrupt genome integrity or protein expression. Overall, our findings support the safety and utility of MSTN gene editing to improve livestock productivity for future food security.

**Abbreviations**
ABC: ammonium bicarbonate
ACN: acetonitrile
ALLERCATPRO: Allergenicity Prediction Program
Alt: alternate allele
BCA: bicinchoninic acid
BWA: Burrows‐Wheeler Aligner
Cas9: CRISPR‐associated protein 9
CBC: complete blood count
CNV: copy number variation
CRISPR: Clustered Regularly Interspaced Short Palindromic Repeats
DTT: dithiothreitol
FASP: filter‐aided sample preparation
FDR: false discovery rate
GATK: Genome Analysis Toolkit
INDEL: insertion and deletion
IVF: in vitro fertilization
IVP: in vitro production
LC–MS/MS: Liquid Chromatography–Tandem Mass Spectrometry
MAPQ: Mapping Quality
MS: mass spectrometry
MSTN: myostatin
NonRefHom: non‐reference homozygous
OPU: ovum pick‐up
PD: Proteome Discoverer
PE: paired‐end
RefHom: reference homozygous
SDS: sodium dodecyl sulfate
SNP: Single Nucleotide Polymorphism
SPS‐MS3: Synchronous Precursor Selection–MS3
SV: structural variant
SVs: structural variants
TALP: Tyrode's Albumin Lactate Pyruvate
TCA: tricarboxylic acid (cycle)
TF: transferrin
TMT: Tandem Mass Tag
UPLC: ultra‐performance liquid chromatography
WT: wild‐type

## 1 Introduction

By the year 2050, the global population is expected to reach about 10 billion, and it has been reported that we will soon face a worldwide food shortage crisis. In this context of food security, cattle are a very important animal for producing animal protein, providing a significant amount through milk and meat. Although improvements in cattle have been achieved through traditional breeding techniques (such as artificial insemination and embryo transfer), new solutions are needed to address the genetic bottlenecks in superior cattle. One method being utilized for this purpose is gene editing.

Gene editing in livestock offers a solution to challenges that conventional breeding techniques cannot address. This technology enables the introduction of genetic traits that are not naturally found within a breed. For instance, there are cattle with enhanced resistance to mastitis, beta‐lactoglobulin knockout (*BLG* KO), hornlessness, and myostatin knockout (*MSTN* KO) (Carlson et al. 2016; Gim, Kwon, et al. 2022; Wall et al. 2005; Wei et al. 2018). Recently, the US Food and Drug Administration has advanced plans to regulate the gene editing of livestock as food (DRUG 2025). Additionally, there is a global review of food regulations concerning gene‐edited livestock and a few livestock with gene editing have been approved in some country (Wray‐Cahen et al. 2024). Although there has been significant success in specific gene editing in many livestock species, there is a notable lack of analysis regarding the genetic stability of these gene‐edited animals and their effects on subsequent generations (Carlson et al. 2016; Whitworth et al. 2016). Therefore, there is a current need for such analyses and research on guidelines for safety assessments of these animals as food.

A previous study by our group evaluated the long‐term health and genomic stability of transgenic cattle generated using transposon‐mediated gene transfer and expressing exogenous fluorescent markers (Yum et al. 2024). While that research demonstrated the safety of non‐viral transgenesis over a 10‐year period, it focused on animals not intended for food production due to the presence of foreign genes. In contrast, the present study addresses gene‐edited cattle generated via CRISPR (Clustered Regularly Interspaced Short Palindromic Repeats)/Cas9 (CRISPR‐associated protein 9) targeting the MSTN gene, specifically without exogenous gene integration, to evaluate their potential as a sustainable and safe food source.

In previous research, we produced cattle with mutations in the myostatin (*MSTN*) gene (Gim, Kwon, et al. 2022; Gim, Uhm, et al. 2022), known for genetic trait that increase carcass weight. Gene editing of the *MSTN* has been performed in various livestock, including cattle, pigs, goats, and sheep (Li et al. 2020; Proudfoot et al. 2015; Wang et al. 2015). However, studies on the genetic characteristics of these gene‐edited individuals and their offspring are limited. The aim of this study is to conduct a comprehensive genomic and proteomic analysis, including assessments of long‐term health and germline transmission, in cattle produced through gene‐editing technologies. The findings of this research will provide objective data regarding the long‐term safety of cattle generated using gene‐editing methods.

## 2 Materials and Methods

### 2.1 Animals

All experimental animals were Hanwoo ( *Bos taurus* ) cattle maintained at the Gyeongsangbuk‐do Livestock Research Institute (Yeongju, Republic of Korea). The *MSTN*‐mutant cohort consisted of two F0 founders (F0‐1, male, born 26 February 2020; F0‐2, female, born 10 April 2020) and four F1 offspring (F1‐1, female, born 6 May 2022; F1‐2, female, born 21 September 2022; F1‐3, male, born 21 October 2022; F1‐4, female, born 21 October 2022).

All animals were housed in a sawdust‐bedded stanchion barn under standard Hanwoo husbandry conditions. Animals were individually restrained in stanchion stalls and fed a diet consisting of commercial concentrate and tall fescue ( *Festuca arundinacea* ) as roughage. Nutritional analysis showed that the concentrate contained 90% dry matter, 17.1% crude protein, 3.5% ether extract, 9.7% crude ash, and 5.49% crude fiber. The tall fescue contained 89% dry matter, 5.1% crude protein, 52.1% total digestible nutrients (TDN), 74.5% neutral detergent fiber (NDF), 46.5% acid detergent fiber (ADF), 5.5% crude ash, and 1.2% ether extract. Fresh water was available ad libitum throughout the experimental period. All experimental procedures were conducted in compliance with institutional animal care guidelines under IACUC approval (#106).

### 2.2 Donor Management and Ovum Pick Up (OPU)

Donor heifers were restrained in a cattle crush, and epidural anesthesia was administered using 5 mL of 2% lidocaine (Daihan, DAIHAN Lidocaine). Ovarian follicles were visualized via ultrasonography device (DRAMINSKI, Blue), and OPU was performed by a single trained technician. A 7.5 MHz transducer probe equipped with a follicular aspiration guide (WTA, cat. no. 10283) and an 18G threaded needle (WTA, cat. no 17927) was used to locate and aspirate follicles. Follicular fluid was collected into 50 mL conical tubes, and cumulus–oocyte complexes (COCs) were retrieved under a stereomicroscope. Any remaining debris from the follicular fluid was collected for primary culture.

### 2.3 In Vitro Fertilization and Culture

Motile spermatozoa were isolated using a two‐step Percoll gradient (45% and 90%) centrifuged at 1680 rpm for 15 min at 35°C. To prepare the 45% layer, equal volumes of 90% Percoll and TALP medium were mixed. Following centrifugation, sperm pellets were washed twice with 3 mL of capacitation‐TALP medium and pelleted again. The processed spermatozoa (1–2 × 10^6^ sperm/mL) were incubated with matured oocytes in 50 μL drops of in vitro fertilization (IVF)‐TALP medium, covered with mineral oil (Nidacon, cat. no. NO‐100), and cultured for 18 h at 38.5°C under 5% CO_2_. Following co‐incubation, cumulus cells were removed from presumptive zygotes, which were subsequently cultured in a defined two‐step medium under a gas mixture of 5% O_2_, 5% CO_2_, and 90% N_2_ (Lim et al. 2007; Saadeldin et al. 2011).

### 2.4 Embryo Transfer

On Day 7 after fertilization (estrus = Day 0), a single blastocyst was transferred non‐surgically into the uterine horn of synchronized recipients. TCM‐199 medium supplemented with 10% fetal bovine serum (FBS) was used to store embryos prior to transfer. Pregnancy status was determined by rectal palpation and ultrasonography on Day 35 and monitored regularly thereafter.

### 2.5 Blood Analysis

General health assessments of MSTN‐edited cattle were conducted by veterinarians. For hematological and serum chemistry evaluation, 5 mL of jugular whole blood was collected into ethylenediaminetetraacetic acid (EDTA) tubes. Complete blood counts (Hemavet 950, Drew Scientific) and serum biochemistry (BS‐400, Mindray) were performed according to the manufacturers' protocols.

### 2.6 Mini‐Sequencing

Target regions (~1000 bp) were first amplified using Maxime polymerase chain reaction (PCR) Pre‐Mix i‐StarTaq (Intron biotechnology, cat. 25167). A second round of PCR was performed to reduce amplicon size (~220 bp) and introduce custom indexing sequences. Final PCR amplification added Illumina‐compatible adapters, and libraries were purified using a PCR purification kit (Macherey‐Nagel NucleoSpin, cat. 740609). Sequencing was carried out on a MiniSeq platform (Illumina), and resulting FASTQ files were analyzed using Cas‐Analyze (https://www.rgenome.net/).

### 2.7 Whole Genome Sequencing

Genomic DNA concentration and purity were assessed via Qubit fluorometry and TECAN measurement. Fragmentation was performed using acoustic shearing (Covaris S2), and libraries were constructed with TruSeq DNA Nano kits (Illumina). Average fragment sizes; ~350 bp were validated via TapeStation 4200 (Agilent Technologies) and quantified using the KAPA Library Quantification Kit (Kapa Biosystems, KK4824). Paired‐end sequencing (2 × 150 bp) was conducted on an Illumina NovaSeq6000 platform. Raw BCL files were converted to FASTQ using the bcl2fastq developed by Illumina (https://github.com/brwnj/bcl2fastq).

### 2.8 Variant Calling Analysis

Raw reads were trimmed using Trimmomatic v0.39 (LEADING:10, TRAILING:10, MINLEN:50) (Bolger et al. 2014) and aligned to the *Bos taurus* ARS‐UCD1.2 reference genome using BWA v0.7.17 (Li and Durbin 2010). Aligned SAM files were sorted using SAMtools v1.19.2 (Danecek et al. 2021) and subjected to variant calling via the GATK HaplotypeCaller pipeline. Joint genotyping was conducted using GATK CombineGVCFs and GenotypeGVCFs (Van der Auwera and O'Connor 2020). Variants in mitochondrial DNA, sex chromosomes, or unplaced scaffolds were excluded. High‐confidence variants were filtered using BCFtools v1.21 (depth ≥ 10, VAF ≥ 0.35) (Danecek et al. 2021), and functional annotation was performed using SnpEff v5.2e (Cingolani et al. 2012). Three variant categories were defined as follows. Type I variants were those in which alternate alleles (ALT) were observed only in the F0 animals, while both the wild‐type (WT) and F1 animals displayed reference homozygous (RefHom) genotypes. Type II variants were characterized by the presence of ALT alleles exclusively in the F1 animals, whereas the WT and F0 animals showed RefHom genotypes. Lastly, Type III variants were defined as those in which ALT alleles were detected in both the F0 and F1 animals, but not in the WT animals, which remained RefHom at those loci.

### 2.9 Identification of Structural Variants (SVs) and Copy Number Variations (CNVs)

Structural variants (SVs) including deletions, duplications, inversions, and translocations were identified using Delly v1.2.6 (Rausch et al. 2012). SV filtering was performed using BCFtools (PRECISE = 1; PE ≥ 20; MAPQ ≥ 60) (Danecek et al. 2021). Copy number variants (CNVs) were called using 10 kbp window‐based normalization with Delly (Rausch et al. 2012). Genomic variants including SNPs, INDELs, SVs, and CNVs were visualized using shinyCircus v2.0 (Wang et al. 2023).

### 2.10 Tissue Sampling and Storage

Skeletal muscle samples were obtained from the *Longissimus dorsi* muscle of three wild‐type Hanwoo cattle (22–24 months of age), one *MSTN*‐mutant F0‐2 (22 months of age), and one *MSTN*‐mutant F1‐2 calf (postnatal day 2). Prior to biopsy, animals were sedated by intramuscular administration of xylazine hydrochloride (Rompun; 1 mL/100 kg body weight), and local anesthesia was achieved by subcutaneous infiltration of 2% lidocaine (2–3 mL per site). Animals were restrained in a cattle crush, and excisional biopsy (3–5 cm incision) was performed aseptically over the dorsal *Longissimus dorsi* region using a sterile scalpel. Tissue cores (1–3 g) were excised, immediately snap‐frozen in liquid nitrogen, and stored at −80°C until further processing.

### 2.11 Tissue Lysis and Protein Digestion

Frozen Longissimus dorsi tissue was pulverized using a CP02 cryoPREP Automated Dry Pulverizer device (Covaris, Woburn, MA). The powdered tissues were resuspended in lysis buffer (4% sodium dodecyl sulfate, 50 mM ammonium bicarbonate) and sonicated using a Q800R sonicator (Qsonica, Newtown). The protein concentration was determined using the Pierce bicinchoninic acid (BCA) Protein Assay Kit (Thermo Fisher Scientific, Waltham, MA). Following lysis, samples were processed using the filter‐aided sample preparation (FASP) protocol with minor modifications. Briefly, 1,4‐dithiothreitol was added to each lysate at a concentration of 20 mM and incubated for 1 h at 37°C. Samples were loaded onto a Microcon centrifugal filter (Millipore, Burlington, MA) and centrifuged at 14 000 *g* for 20 min. The samples were washed three times with FASP‐urea buffer (8 M urea in 50 mM ammonium bicarbonate [ABC]). Next, 200 μL of 80 mM iodoacetamide in 50 mM ABC buffer was added to the filter and incubated for 1 h at 37°C. Subsequently, the samples were washed with FASP‐urea buffer and twice with 50 mM ABC in four times. Trypsin (50:1 w/w, Thermo Fisher Scientific) was added to each sample and incubated overnight at 37°C. Following digestion, the filtrate was collected by centrifugation at 14 000 *g* for 20 min and evaporated to dryness using a concentrator plus system (Eppendorf, Hamburg). Dried samples were resuspended in 200 mM HEPES at pH 8.5 and the peptide concentration was determined using the Pierce BCA Protein Assay kit.

### 2.12 Tandem Mass Tag (TMT)‐6plex Labeling and Multi‐Dimensional Fractionation

Individual peptide samples were labeled with TMT‐6plex reagent (Thermo Fisher Scientific) according to the manufacturer's instructions. Briefly, peptide digests from each sample was mixed with TMT reagent (4:1 TMT reagent: peptide, w/w) and incubated for 30 min at 25°C. This step was repeated. Subsequently, hydroxylamine was added to 5% to quench the remaining reagent and all samples were pooled. The pooled sample was cleaned using a C18 SPE Tube (Supelco, Bellefonte, PA), and the eluate was dried. Offline mid‐pH reversed‐phase fractionation was performed to increase analytical depth. Peptides were separated using a nanoACQUITY ultra‐performance liquid chromatography (UPLC) system (Waters Corporation, Milford, MA) with an in‐house‐packed column (320 μm i.d. × 55 cm) using 3 μm Jupyter C18 particles (Phenomenex, Torrance). The LC flow rate was 7 μL/min with a 92 min linear gradient ranging from 98% solvent A (10 mM ABC, pH 8) to 40% solvent B (90% acetonitrile with 10 mM ABC). Each fraction was collected every 1 min and automatically concatenated into the final 24 pools using the TriVersa NanoMate robot (Advion Inc., Ithaca). All fractions were dried and resuspended in 10 μL of 25 mM ABC for LC–MS3 analysis.

### 2.13 Synchronous Precursor Selection (SPS) Mass Spectrometry to the Third (MS3) Analysis

LC‐SPS‐MS3 analysis was carried out for each fraction using an Orbitrap Eclipse Tribrid MS (Thermo Fisher Scientific) coupled with a NanoAcquity UPLC equipped with an in‐house packed trap (150 μm i.d. × 3 cm) and an analytical column (75 μm i.d. × 100 cm) using 3 μm Jupiter C18 particles. A linear gradient of solvent A (0.1% formic acid) and solvent B (100% acetonitrile (ACN), 0.1% formic acid) was applied at a flow rate of 300 nL/min as follows: 0–5 min, 2%–5% solvent B; 5–15 min, 5%–10% solvent B; 15–190 min, 10%–30% solvent B; 190–195 min, 30%–80% solvent B; 195–200 min, 80% solvent B isocratic, followed by 35 min of column re‐equilibration. Full MS scans (m/z 375–1575) were acquired at a resolution of 120 k. Higher‐energy collisional dissociation (HCD) fragmentation of precursor ions with a charge of 2–7 was performed. The dynamic exclusion value was set to 60 s. The MS2 scans were acquired at a resolution of 15 k (maximum precursor ion injection time [ITmax] of auto and normalized automatic gain control [AGC] target of 200%). The MS3 scans were acquired at a resolution of 30 k (ITmax of auto, normalized AGC target of 500%).

### 2.14 Proteome Identification and Quantification of MS Datasets

All MS3 datasets were searched using Proteome Discoverer (PD, Thermo Fisher Scientific; ver. 2.4.1.15) against the UniProt *Bos Taurus* reference proteome database (February 2023, 37 508 entries). The assignment of MS2 spectra was performed using the SEQUEST HT algorithm, and the resulting peptide hits were filtered at a maximum 1% false discovery rate (FDR) using the Percolator algorithm. Carbamidomethylation of the cysteine residue and TMT labeling of the lysine residue and peptide N‐terminus were set as static modifications, whereas the oxidation of methionine was set as a dynamic modification. To identify the acetylome, acetylation of lysine residues was additionally set as a dynamic modification. Semi‐tryptic specificity, with up to 2 missed cleavages, was applied. The minimum and maximum peptide lengths were 6 and 144, respectively. Mass tolerances for precursor and fragment ions were set to 10 ppm and 0.6 Da, respectively. Normalized abundances reported by the PD were used for further analysis. The values were filtered to retain proteins at high confidence (maximum 1% FDR at the protein level). In the wild‐type samples, only proteins detected in at least two channels were included. However, in the mutant samples, the 129 N channel was excluded due to differences in pedigree, leaving only two available channels. As a result, proteins detected in at least one of these two channels were included in the analysis. For direct comparisons between wild‐type and mutant samples, only proteins that were detected in all three channels of the wild‐type group and both channels of the mutant group were retained.

### 2.15 Identification of Differentially Expressed Proteins

Protein abundance values were derived from TMT reporter ion intensities normalized within Proteome Discoverer v2.4 using total peptide amount normalization across all TMT channels. Average abundance values were calculated separately for the wild‐type group (WT; channels 126, 128 N, 130 N; *n* = 3) and the *MSTN*‐mutant group (MT; channels 127 N and 131; *n* = 2), and a per‐protein fold change (FC) was computed as the ratio of MT average to WT average abundance. Given the limited number of *MSTN*‐mutant samples (*n* = 2) and the absence of true biological replication—an inherent constraint of gene‐edited livestock research—the minimum sample size required for formal statistical testing (e.g., Student's *t*‐test or limma‐based differential expression analysis) was not met. Accordingly, protein expression differences were assessed using a fold change‐based descriptive approach, consistent with the exploratory, discovery‐phase nature of this dataset. Proteins with FC ≥ 2.0 were classified as up‐regulated and proteins with FC ≤ 0.5 were classified as down‐regulated, yielding a total of 175 DEPs (136 up‐regulated and 39 down‐regulated; 5.9% of the 2947 quantified proteins). The complete DEP list is provided in Table S10.

### 2.16 Functional Enrichment Analysis

To place the identified DEPs in a biological context, functional enrichment analysis was performed using curated gene set annotations derived from Gene Ontology (GO) Biological Process terms and Kyoto Encyclopedia of Genes and Genomes (KEGG) pathway categories. Gene names were extracted from UniProt entry fields (GN = identifiers) for all quantified proteins. Twelve functional categories were curated to cover major biological processes represented in skeletal muscle, including extracellular matrix (ECM) organization, cytoskeletal organization, sarcomere structure, glycolysis/gluconeogenesis, TCA cycle/oxidative phosphorylation, fatty acid metabolism, amino acid metabolism, ribosome/translation, ubiquitin–proteasome degradation, cell cycle/DNA replication, RNA processing/splicing, calcium signaling, lipid/lipoprotein metabolism, and oxygen transport. Statistical over‐representation of DEPs within each functional category was assessed using Fisher's exact test (one‐tailed), with the full set of 2813 consistently quantified proteins serving as the statistical background. For each category, a 2 × 2 contingency table was constructed comprising: (i) DEPs assigned to the category, (ii) DEPs not assigned to the category, (iii) background proteins assigned to the category, and (iv) background proteins not assigned to the category. Multiple testing correction was applied across all 12 tested categories using the Benjamini–Hochberg (BH) false discovery rate (FDR) procedure. Functional categories with FDR < 0.05 were considered statistically significant. All analyses were performed in Python (v3.12) using the SciPy library. Results are provided in Table S11.

## 3 Result

### 3.1 Establishment of *MSTN* Mutated F1 Cattle With Stable Germline Transmission

In a previous study, *MSTN* mutations were successfully introduced via microinjection of CRISPR/Cas9 mRNA, resulting in F0‐2 with a 99.9% mutation rate and F0 males with a 10.5% mutation rate (Gim, Kwon, et al. 2022). The stable germline transmission of the *MSTN* mutated trait was confirmed in subsequent analysis (Gim, Uhm, et al. 2022). To enhance offspring production beyond conventional breeding, ovum pick‐up (OPU) was performed on the F0‐2, and in vitro fertilization was carried out using WT semen. Across 13 additional OPU‐IVF trials, 141 oocytes were collected, yielding 20 blastocysts under in vitro culture conditions. This supports the reproducibility and efficiency of *MSTN* mutated embryo production (Gim, Uhm, et al. 2022) (Table S1). Of these 20 blastocysts, 11 were transferred into 11 surrogate cows, with five pregnancies confirmed by ultrasound on Day 35. Ultimately, four calves were born, all heterozygous for the same *MSTN* mutation identified in the F0‐2, as confirmed by mini‐ and whole‐genome sequencing. One calf (F1‐2) died due to intestinal obstruction on the second day post‐birth. Annual blood test was conducted as part of routine health monitoring for both F0 and F1 animals. No significant abnormalities were observed, and the animals are currently growing without health issues (F0‐2: 56 months old; F1‐1: 31 months; F1‐3 and F1‐4: 25 months) (Table 1). To confirm germline transmission from the F1 generation (F1‐3), four IVP (in vitro production) experiments were performed using semen from the *MSTN* F1‐3 bull. Out of 194 oocytes, 36 blastocysts were produced (19.1% ± 13.0%). From these, 27 blastocysts were randomly selected for *MSTN* mutation analysis, and 18 (63.3% ± 14.1%) were found to carry the *MSTN* mutation.

**TABLE 1:** Blood analysis result in MSTN mutated cattle.

Blood parameters	References	MSTN‐mutant cattle			
		F0‐2 (56‐month‐old)	F1‐1 (31‐month‐old)	F1‐3 (25‐month‐old)	F1‐4 (25‐month‐old)
WBC	4.0~12.0 (10^3^/mm^3^)	10.0	9.0	9.4	11.4
RBC	5.0~10.0 (10^3^/mm^3^)	7.04	7.57	8.06	6.38
HGB	8.0~15.0 (g/dL)	11.2	11.4	11.9	10.0
HCT	24.0~46.0 (%)	36.9	36.7	39.2	32.8
PLT	200~800 (10^3^/mm^3^)	236	364	472	383
AST	53~162	68	46	67	57
ALP	77~265	68	130	188	146
BUN	10~25	15.9	10.7	12.1	14.5
Creatinine	0.4~2.0	2.1	1.3	1.6	1.7
Total protein	7.2~9.0	7.6	6.4	6.6	7.0
Albumin	3.2~4.2	3.5	3.4	3.2	3.6
Ca (mg/dL)	8.3~10.4	10.4	10.28	10.76	10.71
P (mg/dL)	4.2~7.7	4.94	4.92	6.97	6.28
Glucose	31~77	38	78	60	69
Total bilirubin	0.01~0.5	0.09	0.05	0.07	0.03

### 3.2 Detection of Genetic Variants, SVs, and CNVs in *MSTN* Mutated Cattle

**FIGURE 1:**
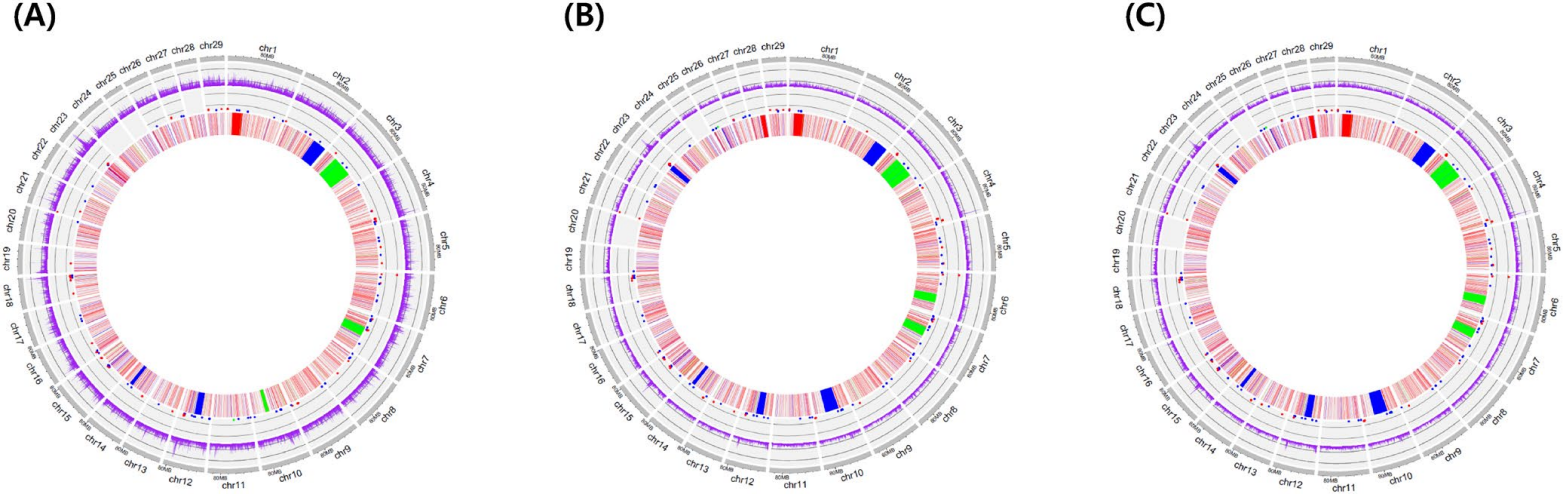
Overview of genomic variation in cattle. An overview of genomic variation in cattle, with the following layers depicted from outer to inner: (1) chromosomes (chr) from chr1 to chr29 are depicted in peripheral gray boxes, based on the reference *Bos Taurus* genome (ARS UCD1.2), (2) the number of SNPs per 100‐kb window is represented in a bar plot (purple), (3) CNVs in indicated positions are represented by dots, with colors indicating different CNV levels: CNV > 2 (red), CNV = 2 (green), and CNV < 2 (blue), and (4) SVs are depicted, including deletion (red), inversion (blue), duplication (green), translocation (purple), and insertion (yellow). (A) WT, (B) F0‐2, and (C) F1‐1.

To detect genetic variants, SVs, and CNVs in WT and *MSTN* mutated cattle, we performed whole‐genome DNA sequencing using blood samples from F0‐2 and F1‐1 *MSTN* mutated cattle and 1‐year‐old WT control cattle. High‐quality filtered reads were mapped to the cow reference genome (ARS‐UCD1.2) with an average mapping rate exceeding 99.7%. Paired‐end reads provided a minimum sequencing depth of 30.4‐fold across the entire genome for variant analysis (Table S2). In total, we identified 6 845 674, 6 633 943, and 6 485 857 SNPs in WT, F0‐2, and F1‐1 cattle, respectively. Additionally, 1997, 2248, and 2197 SVs were detected in WT, F0‐2, and F1‐1 cattle, respectively (Figure 1).

### 3.3 Identification of Specific Genetic Variants and SVs in *MSTN* Mutated Cattle

To investigate specific genetic variations in *MSTN* mutated cattle, we analyzed SNPs and INDELs in *MSTN* mutated cattle compared to WT (Table 2). A total of 688 969 and 637 942 SNPs were uniquely identified in F0‐2 and F1‐1 cattle, respectively, while 1 459 285 SNPs were commonly found in both F0‐2 and F1‐1 cattle. Among these SNPs, the effects of non‐reference homozygous (NonRefHom) SNPs were assessed (Table 2). The majority of SNPs specifically found in *MSTN* mutated cattle were classified as having a “MODIFIER” impact, whereas 2 and 18 HIGH‐impact SNPs were identified in F0‐2 and both *MSTN* mutated cattle, respectively (Table S3). For INDELs, 83 844 and 78 121 were unique to F0‐2 and F1‐1 cattle, respectively, while 185 122 INDELs were commonly found in both F0‐2 and F1‐1 cattle (Table 2). Similar to the result of SNP analysis, the predicted effects of NonRefHom INDELs revealed that more than 99% of these variants were categorized as “MODIFIER” impact (Table 3). Additionally, five HIGH‐impact INDELs were specific to F0‐2, while 21 HIGH‐impact INDELs were found in both F0‐2 and F1‐1 cattle (Table S4). These findings indicated that the majority of SNPs and INDELs specifically found in *MSTN* mutated cattle contained “MODIFIER” impact, suggesting that these variants are unlikely to alter gene function significantly.

**TABLE 2:** Statistics of high‐confidence somatic SNP and INDEL in MSTN mutated cattle compared to wild type.

Variant type	Sample	Genotype	Number of SNPs	Number of INDELs
Type I	WT	Ref Hom	688 969	83 844
	F0‐2	Alt		
	F1‐1	Ref Hom		
Type II	WT	Ref Hom	637 942	78 121
	F0‐2	Ref Hom		
	F1‐1	Alt		
Type III	WT	RefHom	1 459 285	185 122
	F0‐2	Alt		
	F1‐1	Alt		

*Note:* Somatic SNP and INDEL were detected using GATK v4.3.0.0 with the ARS UCD1.2 cattle genome as the reference.

**TABLE 3:** The effects of NonRefHom SNPs and INDELs were predicted using SnpEff.

Variant type	Impact type	SNP count (%)	INDEL count (%)
Type I	HIGH	2 (0.093%)	0 (0%)
	LOW	4 (0.0187%)	0 (0%)
	MODERATE	4 (0.0187%)	0 (0%)
	MODIFIER	2131 (99.533%)	657 (100%)
Type II	HIGH	0 (0%)	5 (0.509%)
	LOW	20 (0.681%)	0 (0%)
	MODERATE	12 (0.409%)	0 (0%)
	MODIFIER	2905 (98.91%)	977 (99.491%)
Type III	HIGH	18 (0.006%)	21 (0.065%)
	LOW	1218 (0.454%)	28 (0.087%)
	MODERATE	752 (0.281%)	13 (0.04%)
	MODIFIER	266 041 (99.259%)	32 067 (99.807%)

Next, we identified structural variants (SVs) unique to *MSTN* mutated cattle. A total of 21 and 12 SVs were specifically found in F0‐2 and F1‐1 cattle, respectively, while 479 SVs were commonly found in both F0‐2 and F1‐1 cattle (Table 4). Effect prediction for NonRefHom SVs revealed that most were categorized as “MODIFIER” impact (Table S5). Three HIGH‐impact SVs found in both F0‐2 and F1‐1 cattle were predicted to affect transcripts of non‐essential genes (*ENSBTAG00000048627*, *ENSBTAG00000052953*, *ENSBTAG00000033030*). Our SVs analysis suggests that SVs specific to *MSTN* mutated cattle are unlikely to disrupt gene function.

**TABLE 4:** Statistics of high‐confidence structural variant (SV) in MSTN mutated cattle compared to wild type.

Variant type	Sample	Genotype	Number of SVs
Type I	WT	Ref Hom	21
	F0‐2	Alt	
	F1‐1	Ref Hom	
Type II	WT	Ref Hom	12
	F0‐2	Ref Hom	
	F1‐1	Alt	
Type III	WT	Ref Hom	479
	F0‐2	Alt	
	F1‐1	Alt	

*Note:* Somatic SV were detected using Delly v1.2.6 with the ARS UCD1.2 cattle genome as the reference.

### 3.4 Genotypic Analysis of the *MSTN* Gene in *MSTN* Mutated Cattle and Allergenic Assessment

To identify the genotype of the *MSTN* gene in *MSTN* mutated cattle, we examined INDELs in *MSTN* mutated cattle compared to WT cattle. We found that the F0‐2 had a biallelic mutation in the *MSTN* gene. Specifically, the ALT1 genotype contained a 12 bp in‐frame deletion, while the ALT2 genotype had a 13 bp deletion with a one base pair frameshift, resulting in a premature stop codon (Tables S6, S7; Figure S1). In the F1‐1 *MSTN* mutated cattle, a heterozygous *MSTN* mutation was observed, consisting of the Ref genotype and the ALT1 genotype found in F0‐2, indicating that the ALT1 genotype in F1‐1 was inherited from F0‐2.

To assess the potential allergenic risks of *MSTN* mutated cattle as a new livestock for human consumption, we conducted allergenic analysis of mutated *MSTN* protein using AllerCatPro 2.0 (Nguyen et al. 2022). This analysis indicated that none of the mutated *MSTN* proteins showed associations with known allergens in the protein database. These results suggest that *MSTN* mutated cattle generated through CRISPR/Cas9 system are unlikely to pose additional allergenic risks compared to WT cattle.

### 3.5 Proteomics Analysis of *MSTN* Mutated Cattle

**FIGURE 2:**
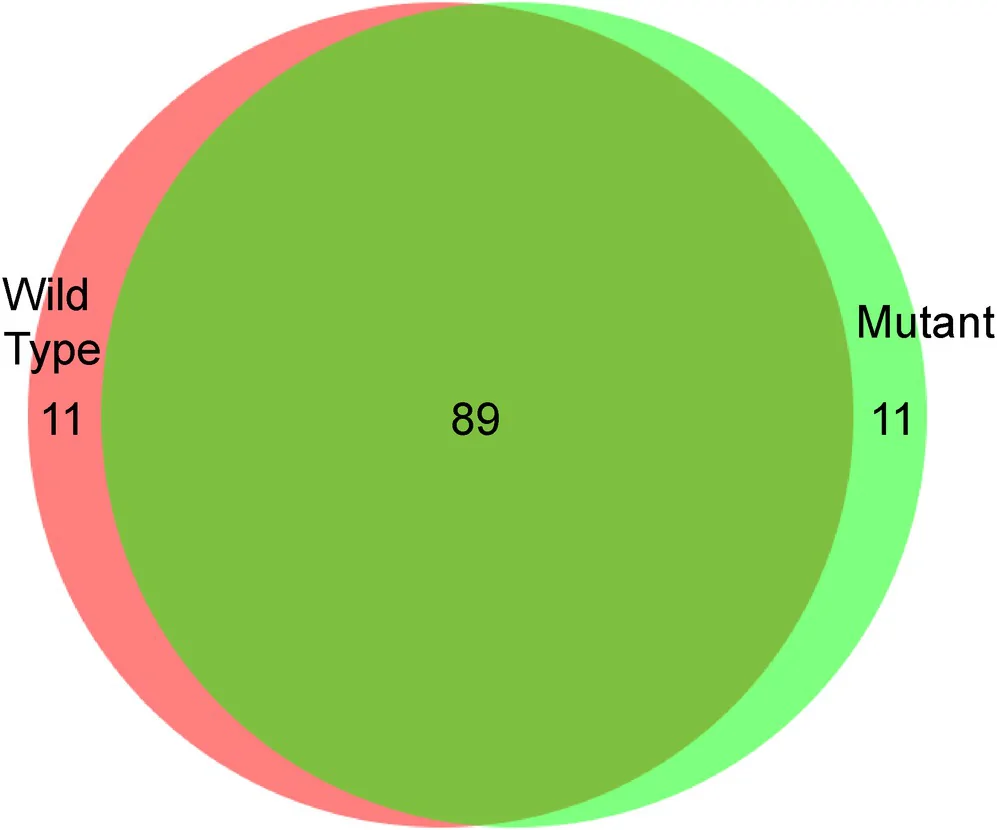
Proteomic similarity between wild‐type and *MSTN*‐mutant cattle. Red circle: Top 100 proteins by average abundance in WT cattle. Green circle: Top 100 proteins by average abundance in *MSTN*‐mutant cattle (F0‐2, F1‐2). Overlap (89): Shared proteins between WT and *MSTN*‐mutant top 100 lists.

To perform in‐depth proteomic profiling of *Longissimus dorsi* tissue obtained from *MSTN* mutated (F0‐2 and F1‐2) and WT cattle, a high‐throughput proteomics workflow based on Tandem Mass Tag (TMT)‐labeled mass spectrometry was employed. As a result, a total of 2960 proteins were detected in both wild‐type and mutant conditions, each quantified across TMT channels with high confidence, applying a false discovery rate (FDR) of ≤ 1% at the protein level. To ensure robustness, proteins included in the dataset exhibited a missing channel count of ≤ 1 in both wild‐type and mutant samples. Compared to the previously reported proteomic dataset of *Bos taurus* *Longissimus dorsi* by Cai et al. (2021), which identified 1943 proteins, our study quantified 2947 proteins, demonstrating the high efficiency and depth of our proteomic profiling approach. Further refining the analysis by including only proteins with no missing channels resulted in a final dataset of 2947 proteins (Table S8), which were consistently detected in both WT and *MSTN*‐mutant cattle. To assess the impact of *MSTN* mutation on proteomic expression patterns, we compared the top 100 proteins ranked by average abundance in WT cattle to the top 100 proteins ranked by average abundance in *MSTN*‐mutant cattle (Figure 2; Table S9). This comparison revealed that 89 proteins were shared between both groups, indicating that the dominant proteomic landscape of skeletal muscle is largely conserved despite the *MSTN* mutation.

To further characterize protein expression differences, fold change (FC)‐based analysis was performed using FC ≥ 2.0 and FC ≤ 0.5 as thresholds for up‐ and down‐regulated proteins, respectively. Given the limited number of mutant samples and the absence of true biological replication—an inherent constraint of gene‐edited livestock research—formal statistical testing was not applicable, and FC‐based descriptive comparison was adopted as the most appropriate approach for this discovery‐phase dataset. Of the 2947 quantified proteins, 136 were up‐regulated and 39 were down‐regulated in *MSTN*‐mutant cattle relative to WT, yielding a total of 175 differentially expressed proteins (DEPs; 5.9% of the total dataset) (Table S10).

Functional annotation of the DEPs revealed statistically significant enrichment in seven biologically coherent categories (Fisher's exact test with Benjamini–Hochberg FDR correction; Table S11). Among up‐regulated DEPs, the most prominently enriched category was extracellular matrix (ECM) organization and collagen remodeling (*n* = 14, FDR < 0.001), including collagen family members (COL1A1, COL1A2, COL6A1, COL6A2, COL6A3, COL12A1, COL14A1), ECM regulators (LOX, FMOD, KERA, POSTN, DCN), and ANGPTL7 (FC = 18.4), the highest fold‐change protein in the dataset. Cytoskeletal and intermediate filament proteins were also significantly enriched (*n* = 11, FDR < 0.001), predominantly comprising up‐regulated hair‐type keratins (KRT89, KRT36, KRT83, KRT31, KRT33A, KRT37, KRT85, KRT86, KRT35), with two members (EPPK1, KRT79) down‐regulated. In addition, several sarcomeric proteins were up‐regulated within the structural muscle/sarcomere category (*n* = 6, FDR = 0.013), including cardiac‐type actin (ACTC1, FC = 5.3), neonatal myosin heavy chain (MYH8, FC = 3.5), myosin light chain 3 (MYL3, FC = 2.0), and myosin light chain 6B (MYL6B), while calponin‐1 (CNN1) and tropomyosin‐1 (TPM1) were down‐regulated. Among down‐regulated DEPs, proteins involved in cell cycle progression and DNA replication were consistently reduced (*n* = 4, FDR < 0.001), including MCM4, PCNA, POLE4, and SMC2. RNA processing and splicing factors were also significantly depleted (*n* = 4, FDR = 0.001), including DDX21, HNRNPA2B1, MTREX, and TRMT112. Additionally, proteins associated with the ubiquitin–proteasome degradation pathway were down‐regulated (*n* = 5, FDR = 0.002), including PES1, URI1, SMC2, POLE4, and MCM4. Lipid and lipoprotein metabolism‐associated proteins showed mixed regulation (*n* = 3, FDR = 0.016), with AFP and AHSG up‐regulated and PLIN1 down‐regulated.

## 4 Discussion

Gene editing technologies, including CRISPR/Cas9, have emerged as powerful tools for generating new livestock breeds with enhanced productivity and improved safety profiles. In particular, targeted disruption of the MSTN gene represents a promising strategy for producing lean beef cattle, and gene‐edited livestock have already been approved for commercialization in several jurisdictions, including Brazil and the United States. In our previous studies, we generated cattle with MSTN mutations via CRISPR/Cas9 without exogenous gene insertion and reported the initial characterization of their phenotypic and genomic features. Although we previously reported the long‐term health and genomic stability of transgenic cattle carrying exogenous fluorescent marker genes using transposon systems (Yum et al. 2024), those animals were not intended for meat production. The current study expands on this work by evaluating MSTN‐edited cattle generated via CRISPR/Cas9 without exogenous gene integration, with direct relevance to meat production and the safety assessment of gene‐edited livestock. In contrast to our prior work, this study incorporates allergenicity assessments and deep quantitative proteomic profiling to comprehensively evaluate the physiological stability and safety of gene‐edited livestock across generations. Notably, in vitro fertilization using frozen semen from an F1 bull (F1‐3) resulted in blastocyst formation, of which approximately 63% exhibited the MSTN mutation, confirming successful germline transmission to the F2 generation.

Taken together, the present study makes several distinct contributions to the field. First, this is a comprehensive longitudinal safety assessment—integrating genomic variant analysis, long‐term health monitoring, allergenicity profiling, and quantitative proteomic characterization—of MSTN‐edited cattle generated via CRISPR/Cas9 without exogenous gene integration. Second, stable germline transmission of the MSTN mutation to the F1 generation was demonstrated, with IVF‐confirmed F2 blastocyst production providing further evidence of heritable and reproducible gene editing. Third, allergenicity profiling of the mutated MSTN protein indicated no association with known allergens, supporting the biological safety of these animals. Fourth, the proteomic analysis achieved the deepest reported coverage of the bovine Longissimus dorsi proteome to date, quantifying 2947 proteins via TMT‐SPS‐MS3 and establishing a high‐confidence reference dataset for future studies on gene‐edited livestock.

While Belgian Blue cattle, a representative example of naturally occurring MSTN‐mutant cattle, grow extremely quickly and have significantly high birth weights, these characteristics have led to well‐documented issues with dystocia (Casas, Bennett, et al. 2004). In contrast, MSTN‐mutant cattle produced through gene editing in this study showed no issues with dystocia across one delivery from F0 and four deliveries from F1. This suggests that the gene‐editing approach employed here may mitigate some of the challenges associated with naturally occurring MSTN mutations.

While the muscle phenotype was clearly observed, it appeared less pronounced macroscopically compared to Belgian Blue cattle. This difference is presumed to result from variation in mutation sites. In Belgian Blue cattle, mutations occur in the mature‐peptide region of Exon 3, whereas the MSTN‐mutant cattle in this study harbored mutations in the pro‐peptide region. Another naturally occurring MSTN‐mutant breed, Limousin cattle, carries a mutation in the pro‐peptide region of Exon 1, and displays a less pronounced phenotype with a relatively lower incidence of dystocia (Casas, Keele, et al. 2004; Casas, Lunstra, and Stone 2004). Consistent with this, a previous study on MSTN‐mutant cattle (Luo et al. 2014) demonstrated that gene editing in the pro‐peptide region of Exon 2 produced cattle with phenotypes visually similar to those observed in the present study. These findings highlight the importance of mutation site selection in determining phenotypic outcomes and suggest that targeted gene editing in the pro‐peptide region may provide a favorable balance between desirable muscle phenotypes and reduced risks of dystocia.

Genomic analysis of MSTN‐mutant cattle revealed that the majority of SNPs, INDELs, and structural variants (SVs) unique to the mutant animals were classified as having a “MODIFIER” impact, indicating minimal predicted functional consequences. Only a small number of high‐impact variants were detected, all associated with non‐essential genes, suggesting a limited risk of disrupting critical biological processes. Routine physical examinations and blood analyses revealed no health abnormalities, and the F0 individual remains in good health at over 5 years of age. These findings support the precision and genomic stability of the CRISPR/Cas9 editing approach employed, and the stable transmission of the MSTN mutation to the F1 generation and F2‐IVF embryos further underscores the reliability and reproducibility of this strategy for introducing desirable productive traits in livestock.

Proteomic analysis of Longissimus dorsi tissue was performed using a TMT‐labeled SPS‐MS3‐based workflow combined with extensive offline fractionation, enabling deep and high‐confidence protein quantification. A total of 2947 proteins were identified and quantified at a false discovery rate (FDR) of ≤ 1% at the protein level, representing a substantial improvement in proteome depth compared to a previously reported *Bos taurus* Longissimus dorsi dataset of 1943 proteins (Cai et al. 2021). To date, proteomic studies specifically focused on MSTN‐mutant livestock muscle remain limited, and previous investigations have predominantly relied on two‐dimensional gel electrophoresis (2‐DE) or label‐free LC–MS/MS approaches, which offer comparatively lower proteome coverage and quantitative precision. Furthermore, whereas prior studies have largely examined naturally occurring double‐muscled breeds such as Belgian Blue cattle, the present study is, to our knowledge, the first to apply deep quantitative proteomics to MSTN‐edited cattle generated by CRISPR/Cas9 without exogenous gene integration, providing a novel proteomic reference dataset directly relevant to the safety evaluation of gene‐edited livestock. Notably, 89 of the top 100 most abundantly expressed proteins were shared between wild‐type and MSTN‐mutant samples, indicating that MSTN mutation does not lead to major alterations in the global proteomic landscape of skeletal muscle under steady‐state conditions. This supports the interpretation that the phenotypic consequences of MSTN mutation may arise not from broad proteomic remodeling, but rather from more targeted modulation of specific pathways or post‐translational mechanisms.

To further characterize protein expression differences, fold change‐based analysis was performed using FC ≥ 2.0 and FC ≤ 0.5 as thresholds for up‐ and down‐regulated proteins, respectively. Of 2947 quantified proteins, 136 were up‐regulated and 39 were down‐regulated in *MSTN*‐mutant cattle relative to WT, yielding a total of 175 DEPs (5.9% of the total dataset). Functional enrichment analysis using Fisher's exact test with Benjamini–Hochberg FDR correction identified seven significantly enriched categories (FDR < 0.05; Table S11). Among up‐regulated DEPs, the most prominently enriched functional category was ECM organization and collagen remodeling (*n* = 14, FDR < 0.001), including multiple collagen subunits (COL1A1, COL1A2, COL6A1, COL6A2, COL6A3, COL12A1, COL14A1, DCN), the collagen cross‐linking enzyme LOX, ECM modulators FMOD, KERA, and POSTN, and ANGPTL7 (FC = 18.4), the highest fold‐change protein in the dataset. The up‐regulation of ECM‐associated proteins is consistent with the known role of MSTN as a regulator of connective tissue remodeling in skeletal muscle, as MSTN has been demonstrated to directly modulate collagen expression via the Smad2/3 signaling pathway (Mendias et al. 2008, 2006). However, MSTN‐null mice have been reported to show reduced intramuscular collagen content (Mendias et al. 2006), contrasting with the collagen up‐regulation observed in the present study. This discrepancy may reflect species differences between rodent and bovine models, or differences between complete knockout and heterozygous/pro‐peptide region mutation, and warrants investigation in future studies. Cytoskeletal and intermediate filament proteins were also significantly enriched (*n* = 11, FDR < 0.001), predominantly comprising up‐regulated hair‐type keratins (KRT89, KRT36, KRT83, KRT31, KRT33A, KRT37, KRT85, KRT86, KRT35), with two members down‐regulated (EPPK1, KRT79). The prominent enrichment of keratins may partially reflect contributions from connective tissue components within the sampled muscle tissue rather than intrinsic myocellular changes, and should be interpreted with caution. Within the structural muscle/sarcomere category (*n* = 6, FDR = 0.013), several proteins were up‐regulated, including cardiac‐type actin (ACTC1, FC = 5.3), neonatal myosin heavy chain (MYH8, FC = 3.5), myosin light chain 3 (MYL3, FC = 2.0), and myosin light chain 6B (MYL6B), while calponin‐1 (CNN1) and tropomyosin‐1 (TPM1) were down‐regulated.

Among down‐regulated DEPs, proteins involved in cell cycle progression and DNA replication were consistently and significantly reduced (*n* = 4, FDR < 0.001), including MCM4, PCNA, POLE4, and SMC2. This pattern is consistent with a shift from myocyte hyperplasia toward hypertrophy as the predominant growth mechanism in *MSTN*‐mutant muscle, as *MSTN* is known to promote cell cycle arrest through Smad2/3‐mediated pathways, and its disruption has been associated with enhanced muscle fiber hypertrophy (Rodriguez et al. 2014; Sartori et al. 2009). RNA processing and splicing factors were also significantly down‐regulated (*n* = 4, FDR = 0.001), including DDX21, HNRNPA2B1, MTREX, and TRMT112, possibly reflecting reduced transcriptional activity associated with decreased cell proliferation. Additionally, proteins associated with the ubiquitin–proteasome degradation pathway were significantly depleted (*n* = 5, FDR = 0.002), including PES1, URI1, SMC2, POLE4, and MCM4, suggesting reduced proteasomal turnover in *MSTN*‐mutant muscle. The down‐regulation of CNN1 (calponin‐1) and TPM1 (tropomyosin‐1) within the sarcomere category is consistent with a shift in fiber‐type composition toward fast‐twitch (Type II) fibers, a well‐documented phenotypic consequence of *MSTN* deficiency in cattle (Girgenrath et al. 2005; Hennebry et al. 2009).

The findings of the present study have direct implications for the practical application of MSTN gene editing in livestock breeding programs. The absence of major genomic instability, the stable germline transmission across generations, and the conservation of the dominant skeletal muscle proteome collectively support the genomic and physiological safety of CRISPR/Cas9‐mediated MSTN editing as a strategy for producing lean beef cattle. As regulatory frameworks for gene‐edited livestock continue to evolve globally, the comprehensive safety data provided by this study may contribute to evidence‐based regulatory evaluation of MSTN‐edited cattle as a sustainable livestock production system. Furthermore, the demonstration of stable germline transmission and the establishment of a deep proteomic reference dataset provide a scientific foundation for the selective breeding and further development of MSTN‐edited Hanwoo cattle.

Several limitations of the present study warrant consideration. First, the proteomic comparison was conducted with only two *MSTN*‐mutant samples (*n* = 2), reflecting the inherent constraints of cohort size in gene‐edited livestock research, where the production and maintenance of gene‐edited animals imposes practical limitations on biological replication. Accordingly, the two mutant samples do not constitute biological replicates in the conventional sense: the F0‐2 (22 months of age) and F1‐2 calf (postnatal day 2) differ not only in generation and heterozygosity—the F0‐2 carrying a biallelic mutation and the F1‐2 carrying a heterozygous ALT1 mutation—but also substantially in age. As skeletal muscle proteome composition undergoes well‐documented developmental changes, including isoform transitions in myosin heavy chains and shifts in metabolic enzyme expression, this age discrepancy represents a significant confounding variable that precludes direct inter‐individual comparison within the mutant group. In particular, the up‐regulation of fetal‐type proteins including alpha‐fetoprotein (AFP, FC = 8.5), alpha‐2‐HS‐glycoprotein (AHSG, FC = 7.8), and hemoglobin fetal subunit beta (FC = 13.5) is likely attributable to the neonatal age of the F1‐2 sample rather than to the *MSTN* genotype per se. Accordingly, protein expression differences between groups were assessed based on fold change rather than formal statistical inference, and the proteomic findings should be interpreted as exploratory and hypothesis‐generating. Future studies employing matched‐age comparisons with adequately powered, properly replicated cohorts will be necessary to draw definitive conclusions regarding the effect of *MSTN* mutation on the skeletal muscle proteome across developmental stages. Validation of candidate DEPs identified in this discovery dataset and mechanistic investigation of downstream signaling pathways, including the MSTN/Smad axis, are designated as primary objectives of planned follow‐up work.

## 5 Conclusions

This study provides the comprehensive longitudinal safety assessment of MSTN gene‐edited Hanwoo cattle produced via CRISPR/Cas9 without exogenous gene integration. The MSTN mutation was stably transmitted to the F1 generation, and IVF‐based blastocyst production confirmed heritable gene editing to the F2 generation. F1 individuals reached sexual maturity in good health, with no observed dystocia or clinical abnormalities. Whole‐genome sequencing revealed that the vast majority of variants unique to MSTN‐mutant cattle carried minimal predicted functional impact, supporting the genomic stability of the editing approach. Allergenicity profiling indicated no association between the mutated MSTN protein and known allergens. Quantitative proteomic analysis of Longissimus dorsi tissue demonstrated broad conservation of the dominant skeletal muscle proteome, with 89% of the top 100 most abundant proteins shared between mutant and wild‐type animals, further supporting the physiological stability of MSTN gene editing. Collectively, these findings support MSTN gene editing via CRISPR/Cas9 as a safe, precise, and heritable approach for producing lean beef cattle and provide a scientific foundation for the evidence‐based evaluation of the genomic stability, germline heritability, and physiological safety of MSTN‐edited cattle, contributing to the responsible development of gene‐edited livestock for sustainable animal production.

## Author Contributions

G.J. devised the experiment. G.‐M.G., K.‐H.E., and S.‐Y.Y. produced and analyzed embryos for mutation. D.‐J.J. and D.‐Y.K. prepared surrogates and transferred embryos. G.‐M.G. and K.‐H.E. collected the samples and analyzed mutation with B.Y., J.Y., and B.Y.C., which was supervised by J.‐S.K. and D.S. G.‐M.G. merged whole data and prepared the manuscript which was edited by G.J. The author(s) read and approved the final manuscript.

## Funding

This research was supported by the National Research Foundation of Korea (NRF) funded by the Korean government (MSIT) under grant numbers NRF‐2017R1A2B300497215, RS‐2023–00260968, 2710004423, and RS‐2025‐05742968. Additional support was provided by the Korea Institute of Planning and Evaluation for Technology in Food, Agriculture and Forestry (IPET) through the Agricultural and Livestock Technology Commercialization Support Program (RS‐2024‐00402798), funded by the Ministry of Agriculture, Food and Rural Affairs (MAFRA). This work was also supported by the Materials/Parts Technology Development Program (project number: 1415187291, “Development of composite formulation with sustained gene release for the treatment of companion animal sarcopenia”), funded by the Ministry of Trade, Industry and Energy (MOTIE, Korea).

## Ethics Statement

This study was approved by the Institutional Animal Care and Use Committee (SNU‐180403‐1) and performed under the guideline of Seoul National University. All animal practices such as blood collection or embryo transfer were carried out by a veterinarian.

## Conflicts of Interest

The authors declare no conflicts of interest.

## Supporting information


**Figure S1:** Alignment of mutated MSTN proteins in MSTN mutated cattle.

**Table S1:** Competency of MSTN mutated embryo development.
**Table S2:** Summary of short read sequencing results of MSTN mutated and wild‐type cattle.
**Table S3:** Annotation of non‐reference homozymous (NonRefHom) SNP in MSTN mutated cattle compared to wild type using SnpEff. Among NonRefHom SNPs, the effect of HIGH‐impact SNPs were predicted.
**Table S4:** Annotation of non‐reference homozymous (NonRefHom) INDEL in MSTN mutated cattle compared to wild type using SnpEff. Among NonRefHom INDELs, the effect of HIGH‐impact INDELs were predicted.
**Table S5:** Annotation of non‐reference homozymous (NonRefHom) SV in MSTN mutated cattle compared to wild type using SnpEff.
**Table S6:** Annotation of MSTN mutation in MSTN mutated cattle using SnpEff.
**Table S7:** Allergenic analysis of mutated MSTN protein using AllerCatPro 2.0.
**Table S8:** Proteomics alalysis of MSTN mutant cattle.
**Table S9:** Proteomic similarity between wild‐type and MSTN‐mutant cattle.
**Table S10:** All differentially expressed proteins—MSTN mutant vs. WT (Total: 175 | Up: 136 | Down: 39).
**Table S11:** Functional enrichment analysis of differentially expressed proteins in MSTN‐mutant vs. wild‐type Hanwoo cattle.

## Data Availability

The datasets used and/or analyzed during the current study are available from the corresponding author on reasonable request. The whole genome sequencing data generated in this study have been deposited in the NCBI Sequence Read Archive (SRA) under the accession number PRJNA1285069 and are publicly accessible. The mass spectrometry proteomics data have been deposited in the ProteomeXchange Consortium via the PRIDE (Perez‐Riverol et al. 2025) partner repository with the dataset identifier PXD065679.
